# Dislocation‐Mediated Lattice Distortion and Griffiths Phase Behavior in Melt‐Spun Ni_2_MnSb Ribbons

**DOI:** 10.1002/advs.75824

**Published:** 2026-05-22

**Authors:** Kun Wang, Siying Yu, Qizhong Zhao, Jiale Guo, Minxia Fang, Yichuan Tang, Pengqaing Hu, Yin Zhang, Chao Zhou, Kaiyan Cao, Fanghua Tian, Sen Yang

**Affiliations:** ^1^ School of Physics MOE Key Laboratory For Nonequilibrium Synthesis and Modulation of Condensed Matter Xi'an Jiaotong University Xi'an P. R. China

**Keywords:** dislocation, griffiths phase, heusler alloy, local lattice distortion, magnetic phase transitions

## Abstract

The Griffiths phase, characterized by the persistence of local ferromagnetic clusters above the Curie temperature, is a key phenomenon in disordered magnetic systems. There are various mechanisms that can explain its formation, local lattice distortion in Heusler alloys is considered a very critical factor. In this work, the Ni_2_MnSb ribbon samples were prepared by melt‐quenching. The causes of local lattice distortion in the Ni_2_MnSb ribbons were investigated, and their role in Griffiths phase formation was analyzed. Structural analyses reveal that increasing wheel speed progressively elevates dislocation density while enhancing local lattice distortions. Correspondingly, magnetic measurements demonstrate a systematic broadening of the Griffiths phase temperature range. Correlation of dislocation density, strain fields, and magnetic response establishes that dislocation‐induced lattice distortions generate spatially inhomogeneous exchange interactions that stabilize ferromagnetic clusters within the paramagnetic matrix. This work not only offers a practical route for the controllable tuning of the Griffiths phase but also provides fundamental insight into the intrinsic mechanism underlying its formation in Heusler systems.

## Graphical abstract

This figure illustrates how melt‐spinning introduces dislocations into Ni_2_MnSb Heusler alloys. TEM images reveal local lattice distortions and associated strain fields surrounding dislocations at a representative wheel speed. As wheel speed increases, these distortions promote the formation of ferromagnetic clusters within a paramagnetic matrix, progressively broadening the temperature range of the Griffiths phase.

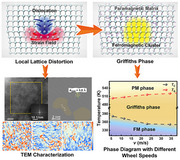

## 1 Introduction

The Griffiths phase (GP) was first proposed in the context of diluted Ising ferromagnets [1] and has since been experimentally observed in a variety of disordered magnetic systems, including both quantum [2, 3, 4, 5] and classical analogues [6, 7, 8]. A defining characteristic of the GP is the emergence of localized spin ordering in the form of ferromagnetic (FM) clusters [9, 10]. These clusters initially form at the onset temperature, *T*_G_, within the global paramagnetic (PM) phase and persist down to the Curie temperature, *T*_C_, of the ferromagnetic transition. Consequently, the inverse magnetic susceptibility exhibits a deviation from the Curie‐Weiss law within the temperature interval *T*_C_ < *T* < *T*_G_ [1].

Several mechanisms have been proposed to explain the formation of the GP in magnetic materials, including: (i) phase separation and magnetic cluster formation [11, 12], characterized by a random distribution of magnetically pure and diluted regions; (ii) a random distribution of exchange interactions [13, 14], which induces spatial variations in the exchange constants, thereby giving rise to localized PM or FM behavior; (iii) thermodynamic inhomogeneity [15], which can result in local magnetic mismatch and spin‐glass behavior; (iv) local lattice distortion [16], as observed in Heusler alloys, where such distortions modify the strength of exchange interactions; and (v) spin‐glass effects [17], wherein mismatches among local magnetic moments promote the GP behavior. In different material systems, one or more of these mechanisms can act in concert to govern the emergence and characteristics of the GP. Therefore, a thorough understanding of the GP necessitates a detailed investigation into the role of these underlying factors in its formation.

Among the mechanisms discussed above, localized lattice distortions have been identified as the origin of the GP in polycrystalline Heusler alloys, as initially proposed by Tian et al. [14]. Owing to their unique magnetic and electronic properties [16], Heusler alloys are promising materials for spintronics and related technologies; notable examples of their functional responses include magnetic shape memory [17, 18, 19], magnetocaloric effects [20, 21], and spin‐valve behavior [22]. Although recent studies on Ni_2_MnSb alloys have suggested that localized lattice distortions contribute to the GP, this hypothesis requires further objective validation.

Melt spinning, a rapid solidification technique, effectively induces grain refinement by generating a high density of nucleation sites [23, 24]. Achieving cooling rates of 10^5^ to 10^7^ K/s [23], this technique inhibits long‐range atomic rearrangement into a perfectly ordered lattice, resulting in high dislocation densities [25, 26]. These dislocations create local strain fields, which give rise to large‐scale lattice distortions that subsequently influence exchange interactions and induce magnetic heterogeneity. In this work, we systematically investigate the influence of wheel‐speed‐tuned dislocation density on lattice distortions and the GP in Ni_2_MnSb Heusler alloys. Our correlative analysis establishes dislocations as a critical microstructural factor controlling the GP formation and stability, and demonstrates a direct processing route for tuning the GP regime. This work provides direct experimental evidence that local lattice perturbations induced by dislocations can modulate electronic correlations, thereby enriching the microscopic understanding of the GP formation in Ni_2_MnSb alloys.

## 2 Results

**FIGURE 1:**
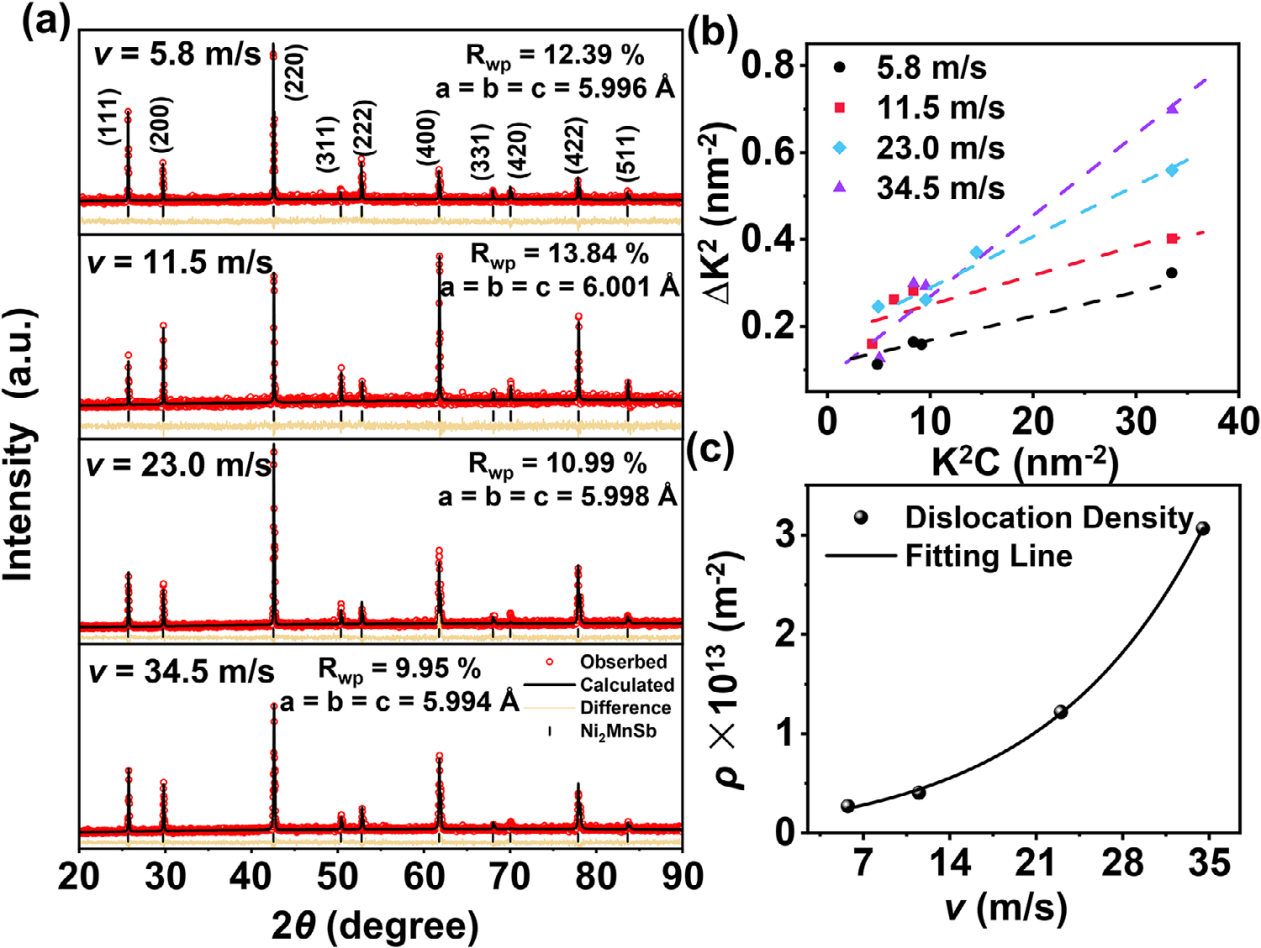
(a) XRD patterns of Ni_2_MnSb ribbons melt‐spun at different wheel speeds (5.8, 11.5, 23.0, and 34.5 m/s); (b) Peak broadening analysis using the modified Williamson–Hall plot; (c) Change in dislocation density *ρ* with quenching wheel speeds for Ni_2_MnSb ribbons.

**FIGURE 2:**
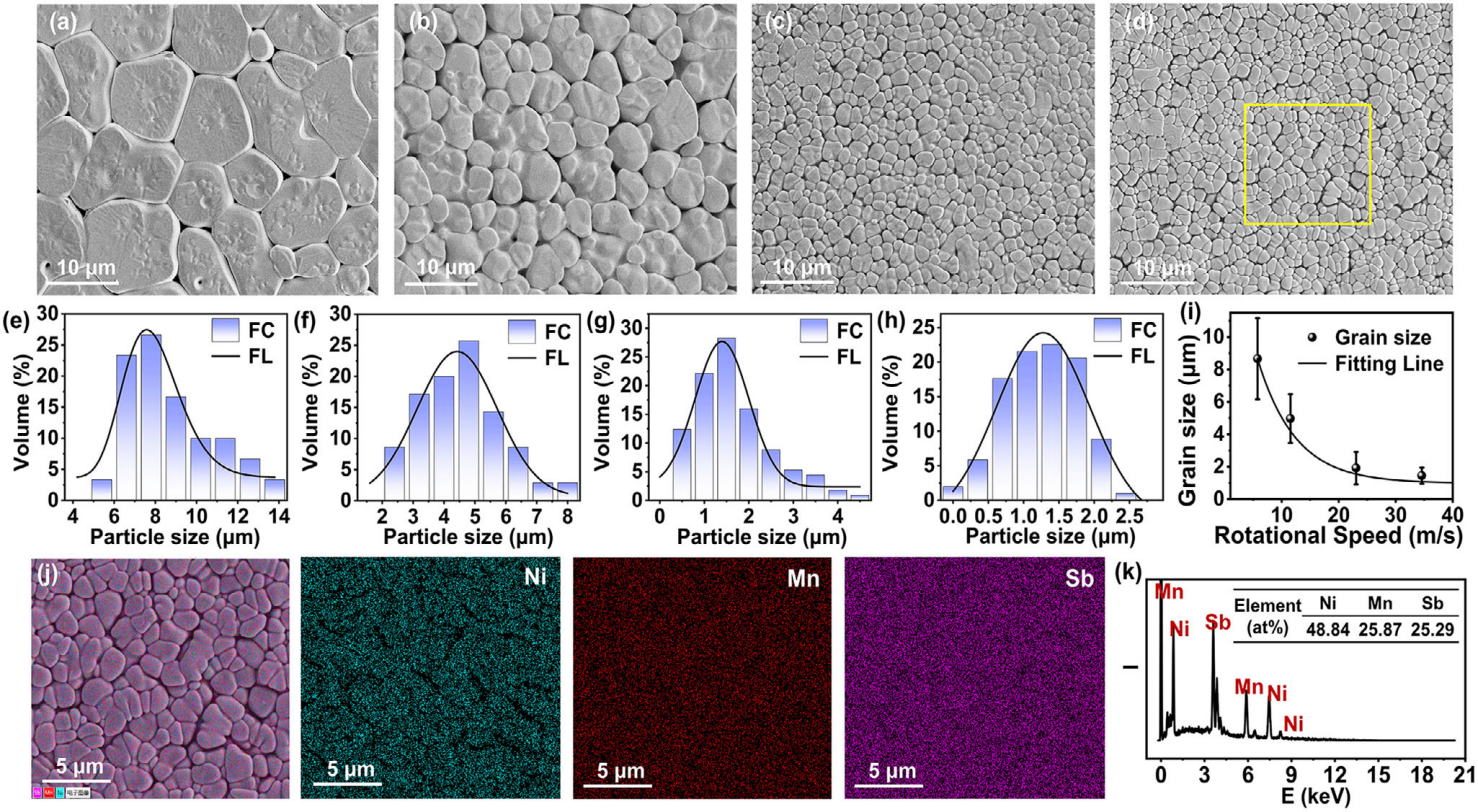
SEM images of melt‐spun Ni_2_MnSb ribbons with different wheel speeds of (a) 5.8 m/s, (b) 11.5 m/s, (c) 23.0 m/s, and (d) 34.5 m/s; (e–h) Grain size measurements from SEM images (a‐d). (i) Grain size as a function of wheel speed. (j) EDS mapping of the region highlighted by the yellow box in the SEM image; (k) EDS spectra from EDS mapping.

Figure 1a presents the x‐ray diffraction (XRD) patterns of the Ni_2_MnSb ribbons prepared at different wheel speeds. The Rietveld refinement was performed assuming a cubic structure with the space group $Fm\bar{3}m$, which yielded a good fit to the experimental data. The refinement results confirm that the alloy crystallizes in the ordered L2_1_ structure, and the corresponding lattice parameters and refined reliability factors are shown in Figure 1. The observed peak broadening is primarily attributed to intrinsic strain within the nanocrystals, originating from crystal imperfections such as point defects, grain boundaries, triple junctions, and stacking faults [27]. The modified Williamson–Hall equation [28] provides a simplified method for using XRD peak broadening to calculate dislocation densities. Assuming that strain broadening is caused by dislocations, the full width at half maximum (FWHM) of the diffraction peaks can be expressed using the modified Williamson–Hall plot as [28]

(1) $$\Delta K≅0.9/D+{\left(\frac{\pi M^{2}b^{2}}{2}\right)}^{1/2}\rho^{1/2}K{\bar{C}}^{1/2}+O\left(K^{2}\bar{C}\right)$$

where *K* = 2 sin *θ*/*λ*, Δ*K* = 2 cos *θ*(Δ*θ*)/*λ*, *θ* is the diffraction angle, Δ*θ* is the FWHM of the diffraction peak, *λ* is the x‐ray wavelength, *D* is the average grain size (as shown in Figure 2e–h), and *b* is the Burgers vector magnitude of the dislocations. For Heusler alloys (e.g., Ni_2_MnAl, Ni_2_MnGa), the slip system is {111}<110>, which is similar to the slip system in face‐centered cubic crystals [29]. Therefore, the magnitude of the Burgers vector *b* depends on the lattice constant *a* and the direction of slip. For the {111}<110> slip system, the formula for the Burgers vector is given in $b=a/\sqrt{2}\approx 4.247$Å. The dislocation density *ρ* represents the average dislocation density. The **O** indicates higher‐order terms in *KC* ^2^, which are ignored in Equation (1). The constant *M* depends on both the effective outer cut‐off radius of dislocations and the dislocation density, and its value has been discussed in the literature [30, 31]. Based on this, the value *M* = 2 is used for all specimens. The value of $\bar{C}$ represents the average contrast factor of the dislocations for a particular reflection. The average contrast factor for different diffraction vectors is defined as

(2) $$\bar{C}={\bar{C}}_{h00}\left[1-qH^{2}\right]$$

where ${\bar{C}}_{h00}$ is a constant related to the elastic constants of the material [32]. *H* ^2^ ​is represented as *H* ^2^ = (*h* ^2^ *k* ^2^ + *k* ^2^ *l* ^2^ + *l* ^2^ *h* ^2^)/(*h* ^2^ + *k* ^2^ + *l* ^2^) where *h*, *k*, and *l* are the Miller indices of each peak. In addition, *q* is a parameter indicating the dislocation character in the samples and can be interpreted as a weighted contribution of edge and screw dislocations, determined by their respective fractions in the sample [32]. The calculation of ${\bar{C}}_{h00}$ is performed by applying the elastic stiffness constant, calculated from first‐principles methods in the literature [33], to the prediction method proposed by Ungár et al. [32]. This calculation results in ${\bar{C}}_{h00}$ = 0.7546. Substituting Equation (2) into Equation (1) yields a linear relationship in terms of *H* ^2^​. The value of *a* is determined by maintaining a linear relationship between *H* ^2^ and (Δ*K* ^2^‐(0.9/*D*) [2])/*K* ^2^. As a result, the value of *q* is obtained from the coefficients of *H* ^2^​ in the linear function [34].

The Δ*K* for each (*h k l*) peak is plotted as a function of *K* ^2^ *C* according to the modified Williamson–Hall equation, as shown in Figure 1b for ribbons with wheel speeds ranging from 5.8 to 34.5 m/s. The plots demonstrate good fits for all curves. The slope (*m*) of the fitted curve is used to calculate the dislocation density from the following equation:

(3) $$\rho =\frac{2m^{2}}{\pi M^{2}b^{2}}$$

where *m* is the slope of the linear fit of the data points on the Δ*K* vs. *K* ^2^ *C* plot. The dislocation densities, calculated using the modified Williamson–Hall equation, are presented in Figure 1c to establish the relationship between dislocation density and wheel speeds for Ni_2_MnSb ribbons. It is clear from Figure 1c that the dislocation density increases from 0.272 × 10^13^ m^−2^ to 3.068 × 10^13^ m^−2^ with increasing wheel speed, as expected. Table 1 presents the FWHM and corresponding diffraction angles for each (hkl) reflection in the XRD patterns of Ni_2_MnSb ribbons melt‐spun at different wheel speeds, together with the corresponding Δ*K* and *K* ^2^ *C* values.

**TABLE 1:** FWHM and diffraction angles (2*θ*) for (hkl) reflections in the XRD patterns of Ni_2_MnSb ribbons melt‐spun at different wheel speeds (5.8, 11.5, 23.0, and 34.5 m/s), together with the corresponding Δ*K* and *K* ^2^ *C* values calculated by the modified Williamson–Hall equation.

Wheel Speed (m/s)	(hkl)	FWHM(2*θ*)	2*θ*	Δ*K* ^2^ (nm^−2^)	*K* ^2^ *C* (nm^−2^)
5.8	111	0.0530(5)	25.668(2)	0.112	4.853
	200	0.0646(5)	29.713(2)	0.164	8.361
	220	0.0698(5)	42.533(2)	0.178	9.149
	400	0.1021(5)	61.736(2)	0.323	33.476
11.5	111	0.0631(5)	25.686(2)	0.159	4.355
	200	0.0847(5)	29.733(2)	0.282	8.371
	220	0.0846(5)	42.563(2)	0.262	6.470
	400	0.1137(5)	61.764(2)	0.401	33.504
23.0	111	0.0783(5)	25.707(2)	0.224	4.942
	200	0.0871(5)	29.763(2)	0.259	8.380
	220	0.0895(5)	42.575(2)	0.371	9.563
	400	0.1343(5)	61.772(2)	0.540	33.505
34.5	111	0.0662(5)	25.743(2)	0.176	5.046
	200	0.0904(5)	29.790(2)	0.322	8.403
	220	0.0971(5)	42.600(2)	0.345	10.053
	400	0.1500(5)	61.795(2)	0.698	33.534

The typical scanning electron microscopy (SEM) images in Figure 2a–d show the microstructures of the free surfaces of the ribbons at different wheel speeds of 5.8 m/s (Figure 2a), 11.5 m/s (Figure 2b), 23.0 m/s (Figure 2c), and 34.5 m/s (Figure 2d), respectively. As expected, the average grain size decreases with increasing wheel speed. The corresponding grain size distributions, statistically analyzed from the SEM images, are displayed in Figure 2e–h, revealing that fine grains with homogeneous sizes are present in all ribbons. These histograms reveal that all ribbons consist of refined grains with a relatively uniform size distribution. Statistical analysis of the grain size distributions indicates no significant intra‐sample variation for ribbons produced at the same wheel speed. A general trend of grain refinement with increasing wheel speed is evident, with the rate of refinement saturating at wheel speeds exceeding approximately 34.5 m/s. The electron energy dispersive spectroscopy (EDS) mapping shown in Figure 2j was collected from the yellow‐boxed selected area in Figure 2d. The elemental maps confirm the uniform spatial distribution of Ni, Mn, and Sb, with no detectable elemental segregation. The EDS spectrum, acquired from EDS mapping, is presented in Figure 2k and confirms that Ni, Mn, and Sb are the principal constituents. The quantitative analysis yields atomic percentages of 48.84% Ni, 25.87% Mn, and 25.29% Sb, which closely match the stoichiometric composition of Ni_2_MnSb.

**FIGURE 3:**
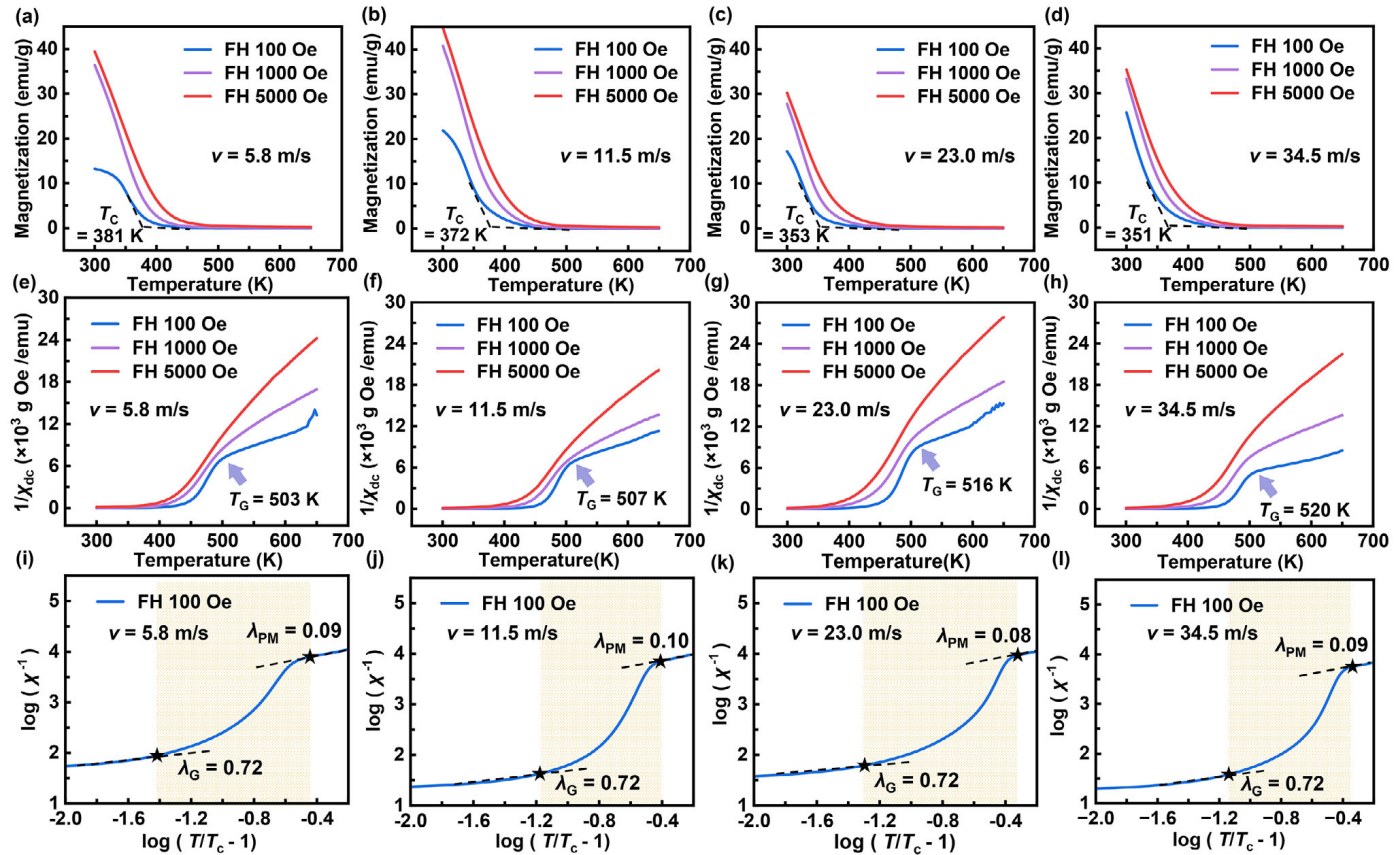
Field‐cooled warming magnetization of melt‐spun Ni_2_MnSb ribbons with different wheel speeds: (a) 5.8 m/s, (b) 11.5 m/s, (c) 23.0 m/s, and (d) 34.5 m/s, measured under different magnetic fields; (e‐h) Temperature dependence of inverse DC susceptibility *χ*−1 under different fields for the ribbons at the corresponding wheel speeds; (i–l) log_10_ *χ* ^−1^ vs log_10_ (*T*/*T*_C_ − 1) plots for melt‐spun Ni_2_MnSb ribbons with different wheel speeds.

The presence of ferromagnetism within the PM matrix in the GP is typically confirmed through macroscopic magnetic measurements. As shown in Figure 3a–d, the M‐T curves for melt‐spun Ni_2_MnSb ribbons at different wheel speeds are plotted during the field‐cooled (FC) process under varying magnetic fields (𝐻_FC_ = 100, 1000 and 5000 Oe). The *T*_C_​ is labeled in the corresponding figure. Figure 3e–h shows the inverse DC susceptibility (𝜒^−1^ = 𝐻/𝑀) vs. temperature plots for Ni_2_MnSb ribbons at different wheel speeds. The inverse susceptibility curve deviates from the Curie–Weiss law with negative curvature above *T*_C_​ (Figure 3a–d). This deviation signals the development of the GP [15], with an onset temperature *T*_G_​ representing the GP transition. As the magnetic field is increased further, the contribution from the PM matrix becomes more significant. A similar downward deviation in 𝜒^−1^(𝑇) from the Curie–Weiss law has been observed in rare‐earth intermetallics and transition metal oxides [35, 36, 37, 38], all attributed to the formation of the GP. For fields exceeding 5000 Oe, 𝜒^−1^(𝑇) becomes nearly linear above *T*_C_​. At temperatures higher than *T*_G_​​, the 𝜒^−1^(𝑇) curves under varying magnetic field strengths do not overlap. This lack of overlap is attributed to the energy required for thermal fluctuations to overcome the spin‐exchange interaction energy and disrupt magnetic order, thereby driving the system into a paramagnetic state. Furthermore, lattice defects can locally alter exchange interactions or magnetic anisotropy, thereby contributing to the field‐dependent variations in the magnetic susceptibility.

The GP can be studied qualitatively by analyzing the power‐law behavior of the inverse DC magnetic susceptibility 𝜒^−1^(𝑇) in the following form [39, 40]:

(4) $$\chi^{-1}\left(T\right)\propto {\left(T-T_{C}\right)}^{1-\lambda }$$

To determine the values of *λ* for the FM and PM states, 𝜒^−1^ ∝ 𝑇/(𝑇‐𝑇_C_) curves are plotted in Figure 3i–l on a log_10_ scale for different wheel speeds and an applied magnetic field of 100 Oe. The slopes of the fitted lines provide the values of 𝜆_G_ ​and 𝜆_PM_ for the GP and PM phase, respectively. These values are consistent with previous studies [14], where 0< 𝜆 < 1 for the GP, while 𝜆 approaches zero for the PM phase. The large value of 𝜆_G_​ suggests the presence of a significant GP in the Ni_2_MnSb alloy. Furthermore, the value of 𝜆_PM_​ < 0.1 establishes that the phase above *T*_G_ is purely paramagnetic, with no evidence of the GP.

**FIGURE 4:**
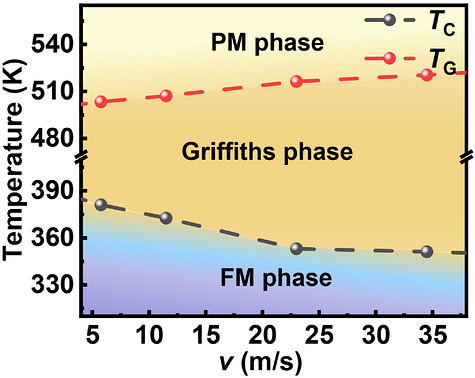
Phase diagram of the melt‐spun Ni_2_MnSb ribbons with different wheel speeds.

As shown in Figure 3, the GP is observed at different wheel speeds. Based on this relationship, the phase diagram (Figure 4) for the melt‐spun Ni_2_MnSb ribbons at various wheel speeds is constructed. Above the transition temperature 𝑇_G_​, thermal motion disrupts the interactions, causing each magnetic moment to be free to rotate, which results in a PM state. For 𝑇_C_ ≤ 𝑇 ≤ 𝑇_G_, as the temperature decreases, nano‐scale ferromagnetic clusters emerge due to local lattice distortions [14]. With the further decrease in temperature, the nano‐scale ferromagnetic clusters gradually grow, and the magnetic interaction increasingly dominates over thermal motion. Below *T*_C_, the system enters a long‐range ferromagnetic state, where all spins align in parallel, and the magnetic moment orientation becomes stable, not changing with time.

The GP regime broadens with increasing wheel speed, a direct consequence of the rising dislocation density in the melt‐spun ribbons. These dislocations generate localized strain fields that perturb exchange interactions and pin ferromagnetic clusters. On a global scale, this lattice distortion weakens the net ferromagnetic exchange and introduces magnetic disorder, thereby suppressing the *T*_C_ by destabilizing long‐range order. Conversely, on a local scale, the strain fields create pockets of pinned moments that act as preferential nucleation sites for ferromagnetic order, enhancing local exchange or anisotropy and stabilizing clusters against thermal fluctuations, thus elevating the *T*_G_. Consequently, increasing the wheel speed concomitantly raises *T*_G_ and lowers *T*_C_, as shown in Figure 4.

**FIGURE 5:**
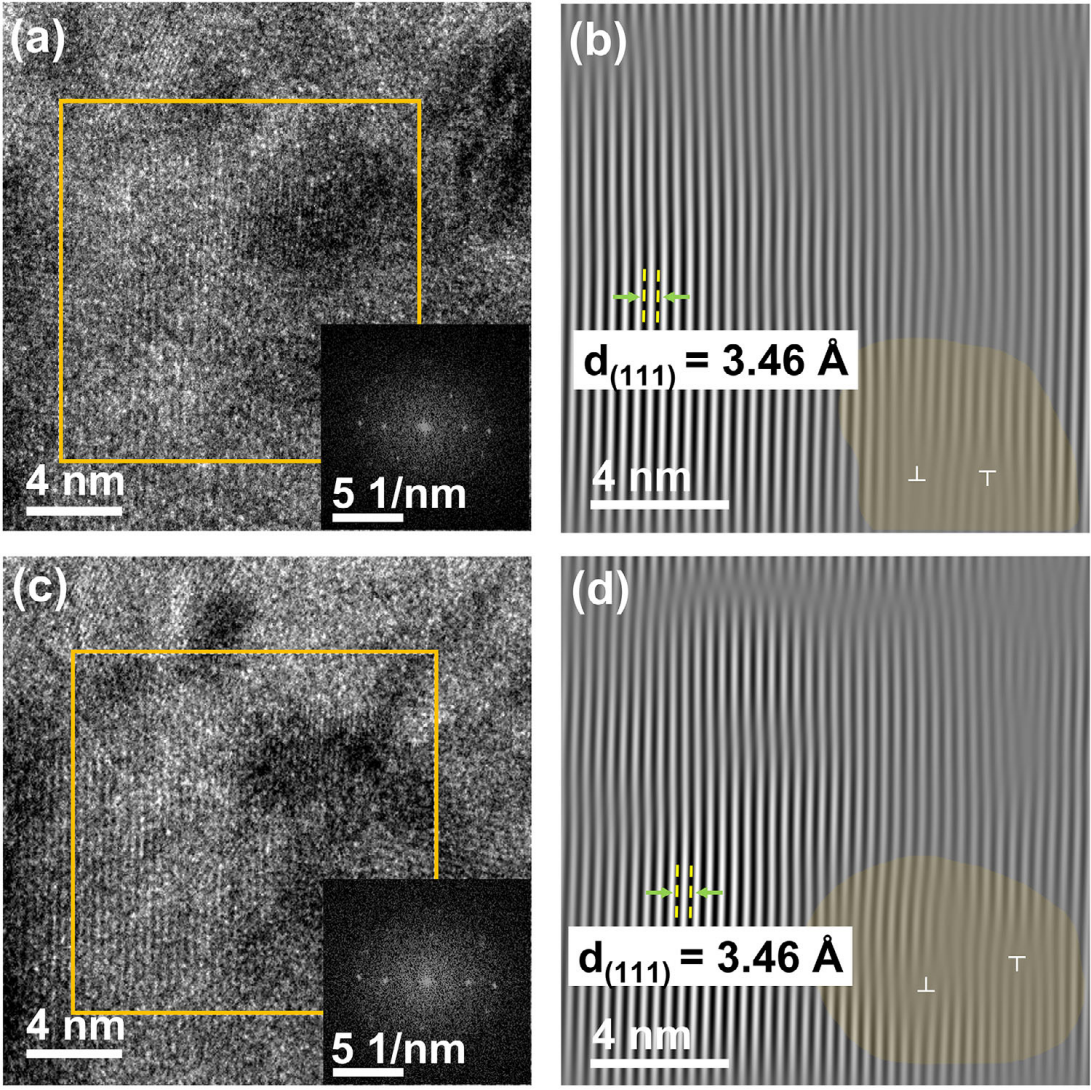
In situ HRTEM images acquired from the identical sample region of Ni_2_MnSb ribbons at 300 and 400 K. (a, c) HRTEM images with FFT insets; (b, d) corresponding IFFT images.

To investigate the influence of temperature fluctuations on local lattice distortion and the resulting GP regime, we obtained in situ high‐resolution transmission electron microscopy (HRTEM) images of Ni_2_MnSb ribbons at 300 and 400 K (both corresponding to a wheel speed of 11.5 m/s), presented in Figure 5a,c, respectively. The corresponding fast Fourier transform (FFT) patterns (insets) confirm the presence of an ordered L2_1_ structure. To probe the local lattice distortion further, we applied an inverse fast Fourier transform (IFFT) filter to selected fundamental reflections, yielding the images shown in Figure 5b,d. Within this temperature interval (i.e., the GP regime), the (111) interplanar spacing in undistorted regions measures approximately 3.46 Å. Comparison of the identical regions reveals that the dislocation densities remain comparable and the spatial extent of the localized lattice distortions exhibits no significant variation between these temperatures.

**FIGURE 6:**
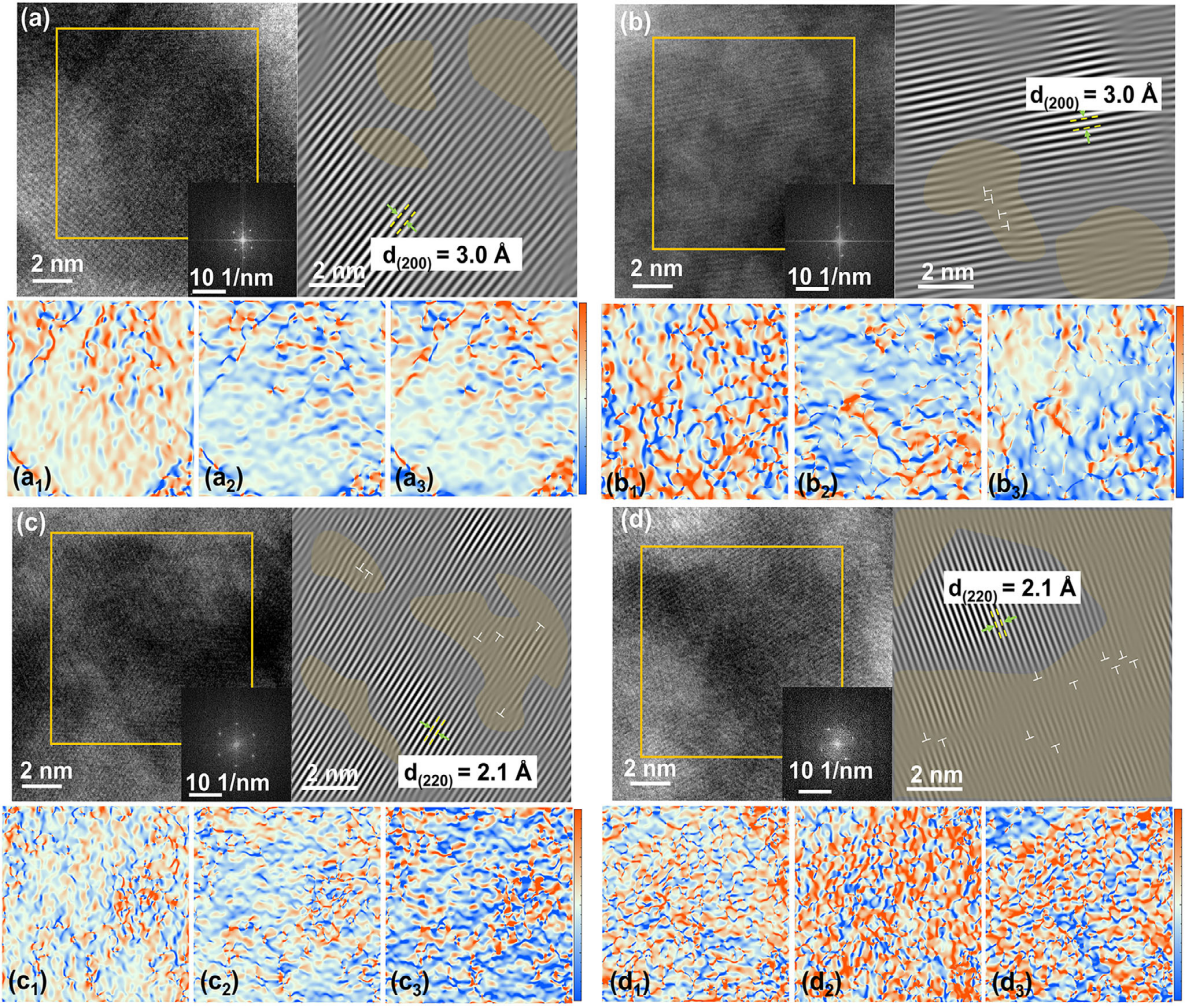
TEM characterizations of melt‐spun Ni_2_MnSb ribbons at different wheel speeds: (a) 5.8 m/s, (b) 11.5 m/s, (c) 23.0 m/s, and (d) 34.5 m/s, at room temperature; Each subfigure includes a HRTEM image, the corresponding FFT pattern extracted from the marked square region, the corresponding IFFT image obtained by selecting the underlying reflections, and the distributions of xx strain (a_1_, b_1_, c_1_, d_1_), yy strain (a_2_, b_2_, c_2_, d_2_), and xy strain (a_3_, b_3_, c_3_, d_3_).

Representative HRTEM images of the Ni_2_MnSb ribbons, synthesized at different wheel speeds, are presented in Figure 6a–d. The corresponding FFT patterns (insets) confirm the presence of an ordered L2_1​_ phase. To assess local lattice distortions, IFFT filtering was applied to selected fundamental reflections. The resulting image, displayed on the right side of the HRTEM image, highlights these distortions. In undistorted regions of the lattice, the measured interplanar spacings are approximately 3.0 and 2.1 Å for the (200) and (220) planes, respectively. As the wheel speed increases, the dislocation density rises, accompanied by an increase in the magnitude of localized lattice distortions. To quantify the magnitude of strain, geometric phase analysis was performed, as shown in Figure 6(a_1‐3_), 6(b_1‐3_), 6(c_1‐3_), and 6(d_1‐3_), using Strain++ software. The strain map clearly indicates that both tensile and compressive strains contribute significantly to the strain in the x and y directions. As the wheel speed increases, the strained regions within the lattice expand, particularly around dislocation cores, underscoring the dominant role of dislocations in governing local lattice distortions. This trend is consistent with the dislocation density quantified from our XRD analysis, providing mutual support from different length scales. The strained regions serve as pinning sites for magnetic moments. This enhances local exchange interactions or anisotropy, thereby stabilizing ferromagnetic clusters against thermal fluctuations. As a result, the GP regime broadens, as observed in Figure 4.

**FIGURE 7:**
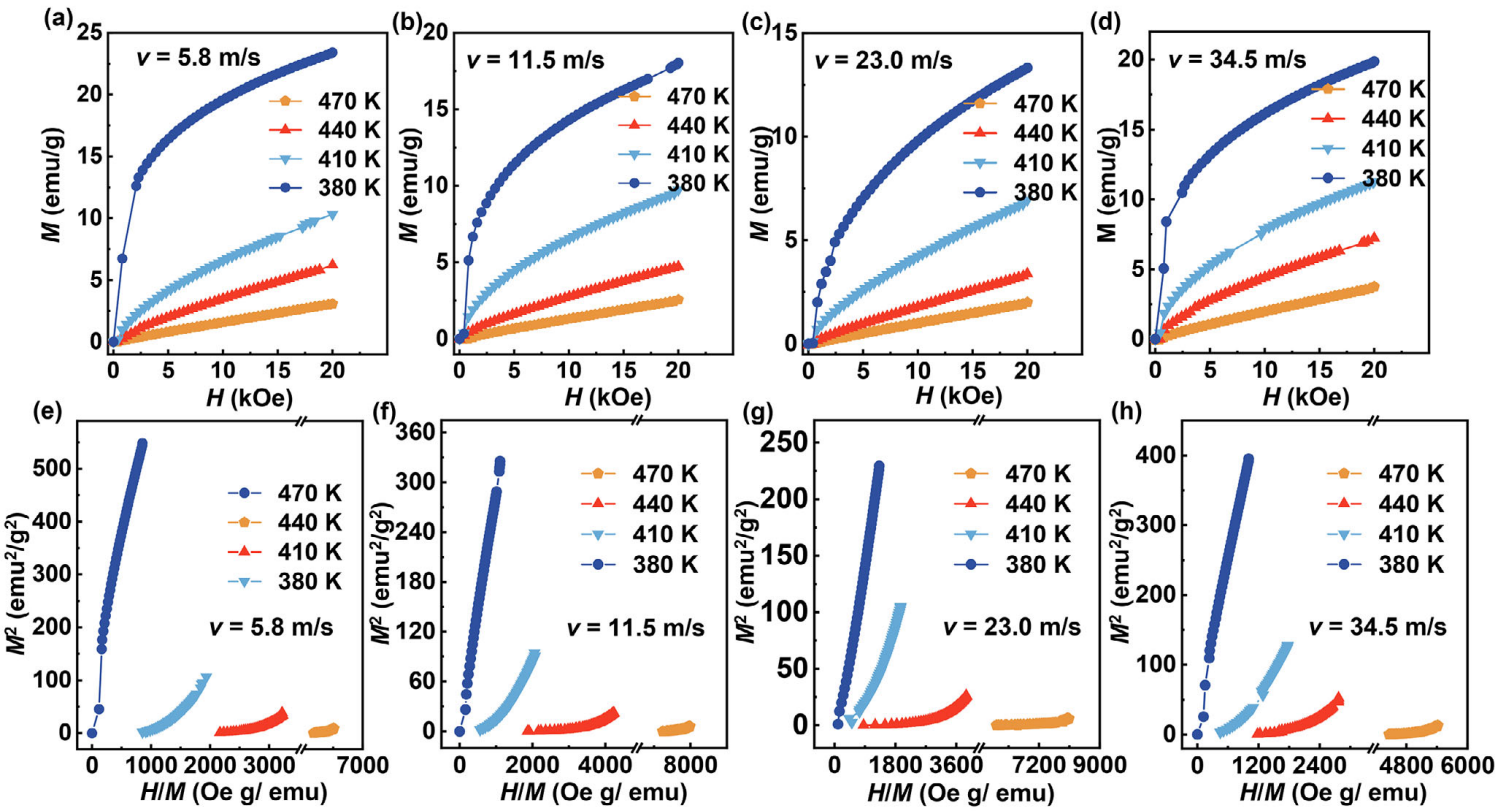
Isothermal magnetization curves for melt‐spun Ni_2_MnSb ribbons fabricated at wheel speeds of (a) 5.8 m/s, (b) 11.5 m/s, (c) 23.0 m/s, and (d) 34.5 m/s, measured in the temperature range (*T*_C_ < *T* < *T*_G_); (e–h), Corresponding Arrott plots derived from the magnetization data.

Arrott plots [41, 42], constructed as *H*/*M* vs. *M* ^2^ plots from experimental isothermal magnetization data (*M* vs. *H*), are commonly used to study ferromagnetic phase transitions. When combined with the Banerjee criterion [43], Arrott plots are frequently employed to analyze and distinguish between first‐ and second‐order phase transitions based on experimental isothermal magnetization data [44]. Figure 7a–d displays the isothermal magnetization of melt‐spun Ni_2_MnSb ribbons at different wheel speeds, with the measured temperatures in the range *T*_C_ < *T* < *T*_G_. The corresponding Arrott plots (Figure 7e–h) consistently exhibit positive slopes. According to the Banerjee criterion [43], the positive slopes identify the magnetic phase transition within the GP regime as second‐order. This finding further implies that the GP in this system does not originate from a structural transformation.

## 3 Conclusions

In summary, we have demonstrated that the temperature window of the GP in Ni_2_MnSb Heusler alloys can be significantly expanded by introducing a high density of dislocations. This work establishes that dislocations, through the localized lattice distortions they generate, serve as the primary mechanism governing the formation and tunability of the GP. XRD analysis quantitatively confirmed the rise in dislocation density with increasing wheel speed, while HRTEM imaging directly visualized the extensive lattice distortion fields associated with these defects. Magnetic susceptibility measurements, along with *M*‐*T* curves, show that the GP temperature range can be effectively controlled by varying the wheel speed. Collectively, these findings provide direct evidence for a causal relationship between dislocation‐induced lattice distortions and the breadth of the GP in Heusler alloys. This study thus offers a fundamental understanding of how engineered microstructural defects can be leveraged to control magnetic inhomogeneity and phase transitions in functional intermetallics, with implications for tailoring Heusler alloys in spintronic applications.

## 4 Experimental Methods

The Ni_2_MnSb alloy ingot was prepared by arc‐melting elemental Ni (99.99%), Sb (99.99%), and Mn (99.9%) under an argon atmosphere on a water‐cooled copper hearth. To ensure chemical homogeneity, the ingots were melted four times. Ribbons were subsequently fabricated from the ingots by single‐roller melt‐spinning under an argon atmosphere, at wheel speeds ranging from 5.8 to 34.5 m/s. The micromorphology and microstructure of the ribbons were characterized using field‐emission scanning electron microscopy (SEM; GeminiSEM 500) and transmission electron microscopy (TEM; JEOL JEM‐2100F). The elemental composition and content of the alloy were analyzed using electron energy dispersive spectroscopy (EDS) and backscattered electron imaging. X‐ray diffraction (XRD) patterns were collected at room temperature using a Bruker D8 ADVANCE Diffractometer. Magnetic properties, including magnetization‐temperature (M‐T) curves and initial magnetization curves, were measured using a superconducting quantum interference device (SQUID) magnetometer (Quantum Design, MPMS‐XL‐5).

## Author Contributions

**Kun Wang**: conceptualization, methodology, software, data curation, investigation, formal analysis, visualization, writing – original draft, writing – review and editing, validation. **Siying Yu**: validation, formal analysis, visualization. **Qizhong Zhao**: investigation, writing – review and editingvisualization, validation, formal analysis. **Jiale Guo**: investigation, visualization, formal analysis. **Minxia Fang**: visualization, formal analysis. **Yichuan Tang**: visualization, investigation. **Penggaing Hu**: visualization, investigation. **Yin Zhang**: investigation, formal analysis. **Chao Zhou**: investigation, formal analysis. **Kaiyan Cao**: formalanalysis, visualization. **Fanghua Tian**: supervision, conceptualization, methodology, data curation, investigation, formal analysis, visualization, project administration, resources, writing – review and editing, funding acquisition. **Sen Yang**: conceptualization, investigation, supervision, resources, project administration, writing – review and editing, funding acquisition, visualization.

## Conflicts of Interest

The authors declare no conflicts of interest.

## Data Availability

The data that support the findings of this study are available in the supplementary material of this article.

## References

[1] R. B. Griffiths , “Nonanalytic Behavior Above the Critical Point in a Random Ising Ferromagnet,” Physical Review Letters 23, no. 1 (1969): 17–19, 10.1103/PhysRevLett.23.17.

[2] R. Hu , K. Wang , H. Ryu , et al., “Electronic Griffiths Phase in the Te‐Doped Semiconductor FeSb_2_ ,” Physical Review Letters 109 (2012): 256401, 10.1103/PhysRevLett.109.256401. 23368482

[3] Y. Xing , H.‐M. Zhang , H.‐L. Fu , et al., “Quantum Griffiths Singularity of Superconductor‐metal Transition in Ga Thin Films,” Science 350, no. 6260 (2015): 542–545, 10.1126/science.aaa7154. 26472763

[4] M. Brando , D. Belitz , F. M. Grosche , and T. R. Kirkpatrick , “Metallic Quantum Ferromagnets,” Reviews of Modern Physics 88, no. 2 (2016): 025006, 10.1103/RevModPhys.88.025006.

[5] Y. Liu , S. Qi , J. Fang , et al., “Observation of In‐Plane Quantum Griffiths Singularity in Two‐Dimensional Crystalline Superconductors,” Physical Review Letters 127, no. 13 (2020): 137001, 10.1103/PhysRevLett.127.137001. 34623853

[6] P. G. Radaelli , D. E. Cox , M. Marezio , S.‐W. Cheong , P. E. Schiffer , and A. P. Ramirez , “Simultaneous Structural, Magnetic, and Electronic Transitions in La_1−x_CaxMnO_3_ With x=0.25 and 0.50,” Physical Review Letters 75, no. 24 (1995): 4488–4491, 10.1103/PhysRevLett.75.4488. 10059921

[7] A. P. Ramirez , P. Schiffer , S.‐W. Cheong , et al., “Thermodynamic and Electron Diffraction Signatures of Charge and Spin Ordering in La_1−x_CaxMnO_3_ ,” Physical Review Letters 76, no. 17 (1996): 3188–3191, 10.1103/PhysRevLett.76.3188. 10060897

[8] I. G. Deac , J. F. Mitchell , and P. Schiffer , “Phase Separation and Low‐Field Bulk Magnetic Properties of Pr_0.7_Ca_0.3_MnO_3_ ,” Physical Review B 63, no. 17 (2001): 172408, 10.1103/PhysRevB.63.172408.

[9] N. Rama , M. S. Ramachandra Rao , V. Sankaranarayanan , et al., “A‐Site‐Disorder‐Dependent Percolative Transport and Griffiths Phase in Doped Manganites,” Physical Review B 70, no. 22 (2004): 224424, 10.1103/PhysRevB.70.224424.

[10] N. Marcano , P. A. Algarabel , L. F. Barquín , et al., “Cluster‐Glass Dynamics of the Griffiths phase in Tb_5−x_LaxSi_2_Ge_2_ ,” Physical Review B 99, no. 5 (2019): 054419, 10.1103/PhysRevB.99.054419.

[11] K. Ghosh , C. Mazumdar , R. Ranganathan , S. Mukherjee , and M. De Raychaudhury , “Structural Correlation With the Griffiths Phase in Disordered Magnetic Systems,” Physical Review B 98, no. 18 (2018): 184419, 10.1103/PhysRevB.98.184419.

[12] A. Pal , P. Singh , V. K. Gangwar , et al., “B‐Site Disorder Driven Multiple‐Magnetic Phases: Griffiths Phase, Re‐Entrant Cluster Glass, and Exchange Bias in Pr_2_CoFeO_6_ ,” Applied Physics Letters 114, no. 25 (2019): 252403, 10.1063/1.5094905.

[13] S. Ubaid‐Kassis , T. Vojta , and A. Schroeder , “Quantum Griffiths Phase in the Weak Itinerant Ferromagnetic Alloy Ni_1−x_Vx,” Physical Review Letters 104, no. 6 (2010): 066402, 10.1103/PhysRevLett.104.066402. 20366837

[14] F. Tian , Q. Zhao , J. Guo , et al., “Griffiths Phase Arising From Local Lattice Distortion and Spin Glass Above the Curie Temperature in Ni_2_MnSb Polycrystalline Heusler Alloy,” Physical Review B 109, no. 22 (2024): 224405, 10.1103/PhysRevB.109.224405.

[15] R. Lu , Y. Ji , Y. Wang , et al., “Evidence for a Griffiths Phase to Cluster Spin Glass Transition in the La_2/3_ Sr_1/3_ (Mn_1‐3x_ Al_2x_ Ti_x_ )O_3_ System,” Advanced Science 11, no. 45 (2024): 2408517, 10.1002/advs.202408517. 39401401 PMC11615757

[16] Y. Sutou , Y. Imano , N. Koeda , et al., “Magnetic and Martensitic Transformations of NiMnX (X = In, Sn, Sb) Ferromagnetic Shape Memory Alloys,” Applied Physics Letters 85, no. 19 (2004): 4358–4360, 10.1063/1.1808879.

[17] “Role of Composition, Site Ordering, and Magnetic Structure for the Structural Stability of off‐stoichiometric Ni_2_MnSb Alloys With Excess Ni and Mn,” Physical Review B 99, no. 6 (2019): 064112, 10.1103/PhysRevB.99.064112.

[18] R. Kainuma , Y. Imano , W. Ito , et al., “Magnetic‐field‐induced Shape Recovery by Reverse Phase Transformation,” Nature 439, no. 7079 (2006): 957–960, 10.1038/nature04493. 16495995

[19] S. Ghosh and S. Ghosh , “Cosubstitution in Ni‐Mn‐Sb Heusler Compounds: Realization of Room‐temperature Reversible Magnetocaloric Effect Driven by Second‐order Magnetic Transition,” Physical Review Materials 4, no. 2 (2020): 025401, 10.1103/PhysRevMaterials.4.025401.

[20] S. Guha , S. Datta , S. K. Panda , and M. Kar , “Room Temperature Magneto‐Caloric Effect and Electron Transport Properties Study on Ni_2.14_Mn_0.55_Sb_1.31_ Alloy,” Journal of Alloys and Compounds 843 (2020): 156033, 10.1016/j.jallcom.2020.156033.

[21] T. Kubota , Z. C. Wen , and K. Takanashi , “Current‐perpendicular‐to‐plane Giant Magnetoresistance Effects Using Heusler Alloys,” Journal of Magnetism and Magnetic Materials 492 (2019): 165667, 10.1016/j.jmmm.2019.165667.

[22] K. Elphick , W. Frost , M. Samiepour , et al., “Heusler Alloys for Spintronic Devices: Review on Recent Development and Future Perspectives,” Science and Technology of Advanced Materials 22, no. 1 (2021): 235–271, 10.1080/14686996.2020.1812364. 33828415 PMC8009123

[23] E. J. Lavernia and T. S. Srivatsan , “The Rapid Solidification Processing of Materials: Science, Principles, Technology, Advances, and Applications,” Journal of Materials Science 45, no. 2 (2010): 287–325, 10.1007/s10853-009-3995-5.

[24] H. Jones , “A Perspective on the Development of Rapid Solidification and Nonequilibrium Processing and Its Future,” Materials Science and Engineering: A 304‐306 (2001): 11–19, 10.1016/S0921-5093(00)01552-5.

[25] Y. J. Lin , S. Mao , Z. Yan , Y. Zhang , and L. Wang , “Melt Spinning Induces Sub‐micrometric/Micrometric Grained Structure and Dislocations in 7075 Al Alloy,” Journal of Alloys and Compounds 651 (2015): 699–704, 10.1016/j.jallcom.2015.08.146.

[26] M. Kowalska , P. Czaja , T. Czeppe , L. Rogal , and M. J. Szczerba , “Anisotropy and Temperature Dependence of Annealing during Mechanical Bending in Ni‐Mn‐Ga‐Based Melt‐Spun Ribbons,” Journal of Materials Engineering and Performance 34, no. 5 (2025): 3800–3810, 10.1007/s11665-024-10524-4.

[27] A. Borbely , “The Modified Williamson‐Hall Plot and Dislocation Density Evaluation From Diffraction Peaks,” Scripta Materialia 217 (2022): 114768, 10.1016/j.scriptamat.2022.114768.

[28] T. Ungár and A. Borbély , “New Approach to Line Profile Analysis,” Applied Physics Letters 69, no. 21 (1996): 3173–3175, 10.1063/1.117951.

[29] W. Everhart and J. Newkirk , “Mechanical Properties of Heusler Alloys,” Heliyon 5, no. 5 (2019): e01578, 10.1016/j.heliyon.2019.e01578. 31080903 PMC6506478

[30] T. Ungár , “Dislocation Densities, Arrangements and Character From X‐ray Diffraction Experiments,” Materials Science and Engineering: A 309‐310 (2001): 14–22, 10.1016/S0921-5093(00)01685-3.

[31] G. Ribárik and T. Ungár , “Characterization of the Microstructure in Random and Textured Polycrystals and Single Crystals by Diffraction Line Profile Analysis,” Materials Science and Engineering: A 528, no. 1 (2010): 112–121, 10.1016/j.msea.2010.08.059.

[32] T. Ungár , I. Dragomir , Á. Révész , and A. Borbély , “The Contrast Factors of Dislocations in Cubic Crystals: the Dislocation Model of Strain Anisotropy in Practice,” Journal of Applied Crystallography 32, no. 5 (1999): 992–1002, 10.1107/S0021889899009334.

[33] S. Ağduk and G. Gökoğlu , “Ab Initio Lattice Dynamics of Ni_2_MnX (X = Sn, Sb) Magnetic Shape Memory Alloys,” Journal of Alloys and Compounds 511, no. 1 (2012): 9–13, 10.1016/j.jallcom.2011.08.092.

[34] T. Shintani and Y. Murata , “Evaluation of the Dislocation Density and Dislocation Character in Cold Rolled Type 304 Steel Determined by Profile Analysis of X‐Ray Diffraction,” Acta Materialia 59, no. 11 (2011): 4314–4322, 10.1016/j.actamat.2011.03.055.

[35] J. Burgy , M. Mayr , V. Martin‐Mayor , A. Moreo , and E. Dagotto , “Colossal Effects in Transition Metal Oxides Caused by Intrinsic Inhomogeneities,” Physical Review Letters 87, no. 27 (2001): 277202, 10.1103/PhysRevLett.87.277202. 11800911

[36] K. Ghosh , C. Mazumdar , R. Ranganathan , and S. Mukherjee , “Griffiths Phase Behaviour in a Frustrated Antiferromagnetic Intermetallic Compound,” Scientific Reports 5, no. 1 (2015): 15801, 10.1038/srep15801. 26515256 PMC4626802

[37] N. Marcano , P. A. Algarabel , J. R. Fernández , et al., “Pressure Dependence of the Griffiths‐Like Phase in 5: 4 Intermetallics,” Physical Review B 102, no. 17 (2020): 174416, 10.1103/PhysRevB.102.174416.

[38] W. Wang , J. Fan , X. Zhong , et al., “Emergence of Griffiths Phase and Exploiting Magnetic Ordering state in the Intermetallic LaCeCo_7_ ,” Journal of Magnetism and Magnetic Materials 529 (2021): 167868, 10.1016/j.jmmm.2021.167868.

[39] C. Magen , P. A. Algarabel , L. Morellon , et al., “Observation of a Griffiths‐Like Phase in the Magnetocaloric Compound Tb_5_Si_2_Ge_2_ ,” Physical Review Letters 96, no. 16 (2006): 167201, 10.1103/PhysRevLett.96.167201. 16712265

[40] A. Ślebarski , J. Goraus , and M. Fijałkowski , “Short‐range Ferromagnetic Correlations in Disordered Fe_2_VGa With Distinct Similarities to the Griffiths Phase,” Physical Review B 84, no. 7 (2011): 075154, 10.1103/PhysRevB.84.075154.

[41] A. Arrott , “Criterion for Ferromagnetism From Observations of Magnetic Isotherms,” Physical Review 108, no. 6 (1957): 1394–1396, 10.1103/PhysRev.108.1394.

[42] A. Arrott and J. E. Noakes , “Approximate Equation of state for Nickel near Its Critical Temperature,” Physical Review Letters 19, no. 14 (1967): 786–789, 10.1103/PhysRevLett.19.786.

[43] B. K. Banerjee , “On a Generalised Approach to First and Second Order Magnetic Transitions,” Physics Letters 12, no. 1 (1964): 16–17, 10.1016/0031-9163(64)91158-8.

[44] L. A. Burrola‐Gándara , C. R. Santillan‐Rodriguez , F. J. Rivera‐Gomez , et al., “Comparison of the Order of Magnetic Phase Transitions in Several Magnetocaloric Materials Using the Rescaled Universal Curve, Banerjee and Mean Field Theory Criteria,” Journal of Applied Physics 117, no. 17 (2015): 17D144, 10.1063/1.4918340.

